# Attitudes and barriers towards deprescribing in older patients experiencing polypharmacy: a narrative review

**DOI:** 10.1038/s41514-023-00132-2

**Published:** 2024-01-23

**Authors:** Michael Robinson, Sophie Mokrzecki, Andrew J. Mallett

**Affiliations:** 1 Townsville Institute of Health Research and Innovation, Townsville University Hospital, Douglas, QLD Australia; 2 College of Medicine and Dentistry, James Cook University, Douglas, QLD Australia; 3 Department of Pharmacy, Townsville University Hospital, Douglas, QLD Australia; 4 Institute for Molecular Bioscience, The University of Queensland, Brisbane, QLD Australia

**Keywords:** Geriatrics, Therapeutics

## Abstract

Polypharmacy, commonly defined as ≥5 medications, is a rising public health concern due to its many risks of harm. One commonly recommended strategy to address polypharmacy is medication reviews, with subsequent deprescription of inappropriate medications. In this review, we explore the intersection of older age, polypharmacy, and deprescribing in a contemporary context by appraising the published literature (2012–2022) to identify articles that included new primary data on deprescribing medications in patients aged ≥65 years currently taking ≥5 medications. We found 31 articles were found which describe the current perceptions of clinicians towards deprescribing, the identified barriers, key enabling factors, and future directions in approaching deprescribing. Currently, clinicians believe that deprescribing is a complex process, and despite the majority of clinicians reporting feeling comfortable in deprescribing, fewer engage with this process regularly. Common barriers cited include a lack of knowledge and training around the deprescribing process, a lack of time, a breakdown in communication, perceived ‘abandonment of care’, fear of adverse consequences, and resistance from patients and/or their carers. Common enabling factors of deprescribing include recognition of key opportunities to instigate this process, regular medication reviews, improving lines of communication, education of both patients and clinicians and a multidisciplinary approach towards patient care. Addressing polypharmacy requires a nuanced approach in a generally complex group of patients. Key strategies to reducing the risks of polypharmacy include education of patients and clinicians, in addition to improving communication between healthcare providers in a multidisciplinary approach.

## What is polypharmacy?

Polypharmacy is most commonly defined as the concurrent use of five or more medications, including prescribed, over-the-counter, traditional, and complementary medicines^1^. Polypharmacy can often be clinically appropriate and beneficial, however, it may still present risks of harm, including adverse drug events, drug-drug interactions, increased risk of hospital admission, non-adherence to treatment regimes, and mortality^1,2^. Patients above the age of 65 are considered a group particularly vulnerable to these risks^2^. This is due to the increased likelihood of experiencing polypharmacy, as well as the change in pharmacological states of medications,, including both pharmacokinetic and pharmacodynamic properties, resulting in variable efficacy of treatment in the aging population^2^.

Polypharmacy has been recognised as an increasing public health challenge worldwide, with rates expected to rise due to an ageing population^1^. Aside from the health impacts, polypharmacy also has an economic impact with an estimated 0.3% of global total health expenditure potentially avoidable with appropriate management of polypharmacy^1^. In Australia, a 2018 study on prescription medications dispensed through the Pharmaceutical Benefits Scheme (PBS) showed that 20.9% of the population experienced polypharmacy, and 3.3% experienced hyperpolypharmacy (≥10 medications)^3^. In patients ≥70 years of age, 45% take ≥5 medications, and 8.3% take ≥10 medications^3^. The most frequently used medications in PBS-eligible Australian patients, located in Australia, experiencing polypharmacy are those prescribed for the cardiovascular, nervous system, and alimentary tract and metabolism indications^3^.

As discussed by Rankin et al. in 2018, the ‘prescriber’s dilemma’ is *“differentiating between ‘many medications’ (appropriate polypharmacy) and ‘too many medications’ (inappropriate polypharmacy)”*. Inappropriate polypharmacy has been described as when the potential harms of a medication outweigh its benefits^4^. A recent report by the Department of Health and Social Care in the United Kingdom estimated that at least 10% of the total primary care prescriptions are not required^5^. Further, a systematic review by Opondo et al. in 2012 found that approximately 20% of prescriptions to elderly patients in primary care are inappropriate^6^.

## How can we address polypharmacy?

Medication reviews, a structured evaluation of an individual’s medicines, are a widely recommended strategy to address polypharmacy^1^. The aim of a medication review is to improve patient outcomes by optimising the use of medicines in a person-centred approach^1^. A Cochrane review by Christensen et al. in 2016 found that medication reviews, characterised as a review of a list of medications with the aim to improve pharmacotherapy by optimising effectiveness and minimising harms and/or costs, can reduce presentations to the emergency department by 27%, although there is no evidence to suggest that medication reviews alone reduce mortality or hospital readmissions^7^. There are also tools that exist to help facilitate the subsequent evaluation of appropriateness of drug therapy, such as the Medication Appropriateness Index^8^.

A strategy to manage polypharmacy that can follow on from medication reviews, is deprescribing. Deprescribing is *“the process of tapering, stopping, discontinuing, or withdrawing drugs, with the goal of managing polypharmacy and improving outcomes”*^1^. Deprescribing has also been defined as *“the systematic process of identifying and discontinuing drugs in instances in which existing or potential harms outweigh existing or potential benefits within the context of an individual patient’s care goals, current level of functioning, life expectancy, values, and preferences.”*^9^ It is often specified as being undertaken under the supervision of a healthcare professional^10^.

Deprescribing is a relatively new term and emerging field of research, with the year of entry in search engines being 2016 (MEDLINE and EMCARE) and 2020 (CINAHL). Other associated terminology includes ‘potentially inappropriate prescribing’ (PIP), and ‘potentially inappropriate medications’ (PIMS). PIMS is commonly determined according to the Beers Criteria, which provides a list of medications to aim to avoid in older patients, if possible^11^. Other tools, such as the Screening Tool of Older Persons’ potentially inappropriate Prescriptions/Screening Tool to Alert to Right Treatment (STOPP/START) have been developed to provide evidence-based practices for over- and under-treatment of medical conditions^12^. A qualitative study in 2020 by Ross et al. described elderly patients acceptance of the paradox of medications being both advantageous and detrimental, by justifying it as *“a personalised medication routine is needed to promote well-being in later life”*, as well as *“the harms associated with medications are externalised to other older adults”*, and *“age-related illnesses are common and therefore seniors need medications to promote health and maintain quality of life”*^13^.

**Fig. 1:**
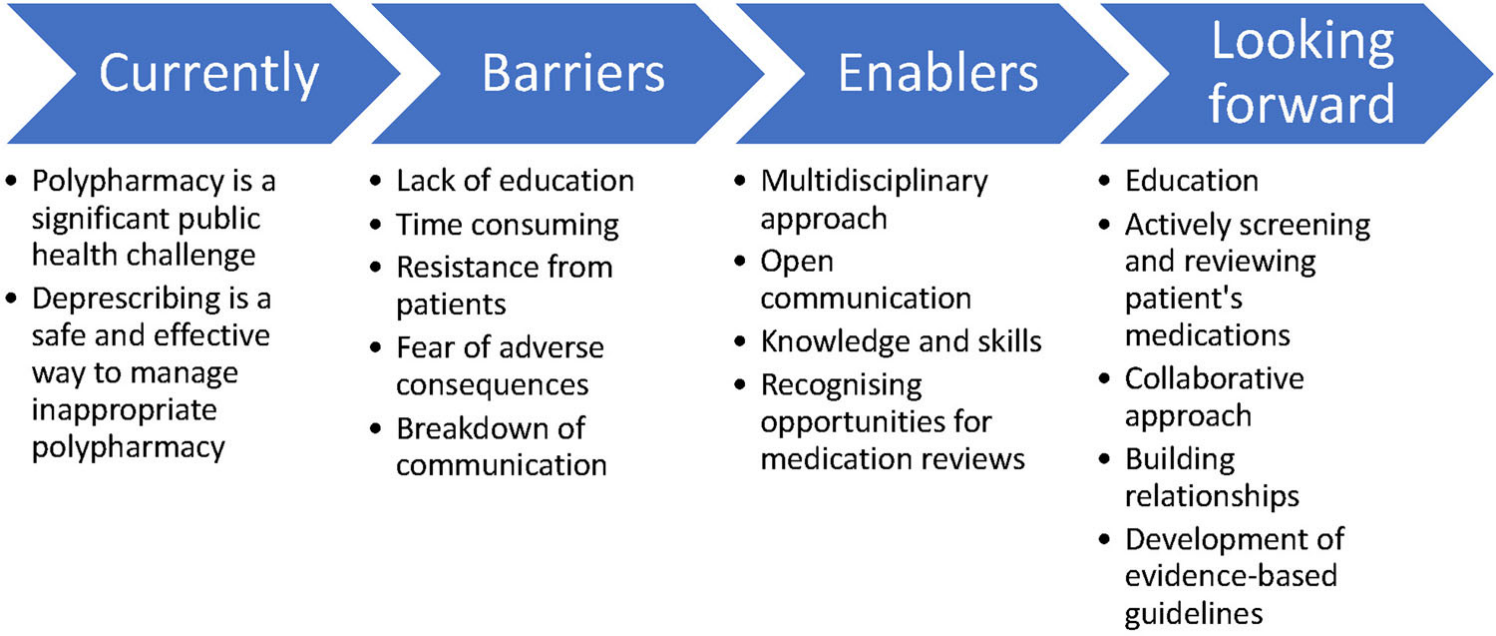
Overview of Deprescribing.

**Fig. 2:**
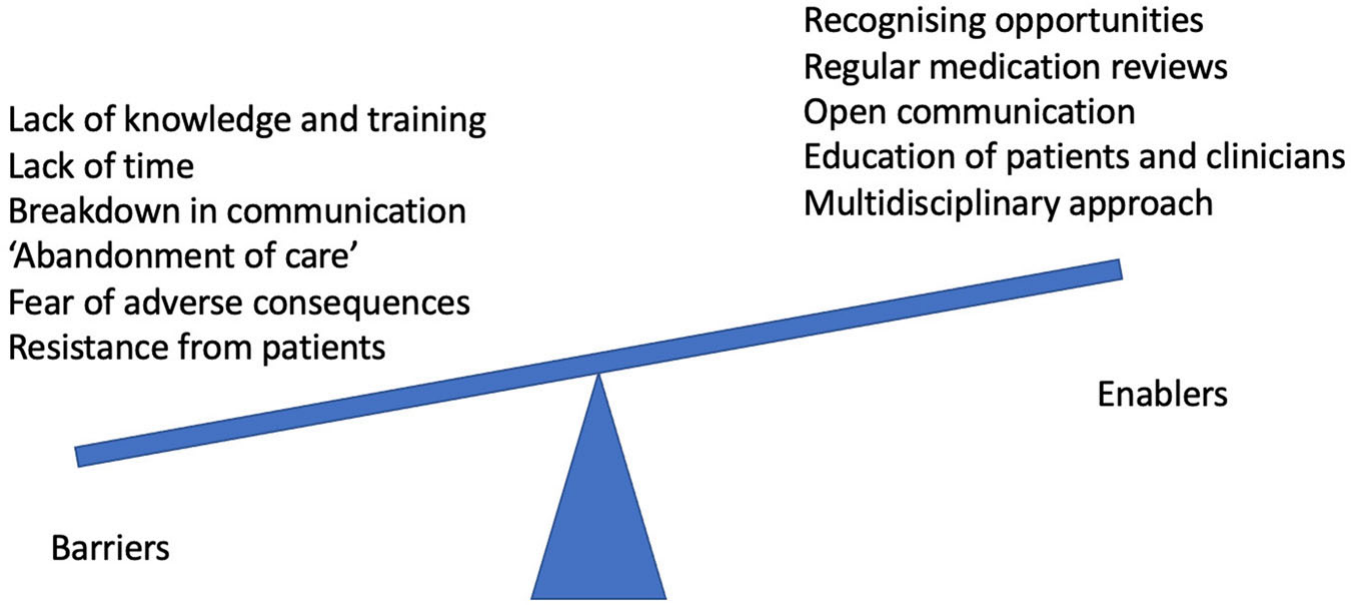
Balance of Barriers and Enablers to Deprescribing.

McDonald et al. in 2022 conducted a cluster randomised clinical trial in Canada where 11,922 older patients experiencing polypharmacy were recruited^14^. This study determined that deprescribing did not have an impact on reducing short-term adverse drug events, although the deprescribing intervention did effectively stop PIMs with no subsequent evidence of increased harm due to the discontinuation of medications. Deprescribing was also noted to have value in avoiding excess cost, waste, and pill burden^14^. A 2021 article by McConeghy et al. described a retrospective cohort study examining a hold of non-essential medications by 64 nursing homes in the United States during the COVID-19 pandemic^15^. In all, 3247 residents had 5297 medications withheld for a median of 60 days, and by the end of the hold, 54% of these medications were permanently ceased^15^. These included probiotics, histamine-2 receptor antagonists, other antihistamines, and statins^15^. This study did not assess if there were any benefits or harms associated with deprescribing (Figs. 1 and 2).

Deprescribing has been demonstrated to significantly reduce mortality when approached in a person-specific manner, and often carries no adverse effects on quality of life or health outcomes^16^. Despite deprescribing having the potential to help safely reduce risks, the problem of inappropriate polypharmacy persists. Deprescribing has been described as currently being performed in a reactive approach (i.e., in response to a clinical trigger), rather than being approached proactively (i.e., in response to reconciling risks)^17^. In order to explore and define current attitudes, barriers, enabling factors, and future directions in deprescribing medications in older patients experiencing polypharmacy, we undertook a semi-structured literature review. We searched Medline, CINAHL, Emcare, and SCOPUS databases for articles from 2012–2022 using MeSH terms and keywords such as deprescribing, drug tapering, polypharmacy, inappropriate prescribing, and aged (see Supplementary Methods).

## Current perceptions towards deprescribing

The causes of inappropriate polypharmacy are numerous, including multiple healthcare providers, lack of understanding of patient’s medical conditions, poor follow-up of patients, poor adherence to medication regimes, and increased use of over-the-counter medications^18^. However, despite the majority of doctors recognising that overuse of medications is a real issue^19–22^, and the reported comfort with potentially deprescribing^4,19,20,23–25^, fewer engage with the process on a regular basis^4,20,22,25^. In contrast, the majority of the patients experiencing polypharmacy feel like the number of medications that they are taking is necessary, although paradoxically most would like to reduce the number of medications prescribed if possible^26–32^.

Deprescribing is viewed as a complex process with a multitude of competing factors^33^. The process is guided by perceptions of risk-benefit ratios, and has been described as an inherently uncertain venture with complex internal reasoning in diverse and complex patients^34^. In addition, a culture that promotes prescribing can also hinder deprescribing medications, in part due to a lack of support and evidence towards deprescribing^35^. From an ethical standpoint, the continuation of a medication by a clinician can be viewed as a passive act, or omission, with minimal responsibility for negative outcomes, whereas the discontinuation of a medication can be viewed as an active and conscious choice, which attaches a larger moral weight to the potential consequences^35,36^. Another perspective to then consider is the allocation of weight on decision-making, for example, is deprescribing an action, or simply the discontinuation, or omission, of a previous prescribing action. One perspective raised by Reeve et al. in 2016 is for clinicians to consider ongoing prescription of medication as an action rather than an omission^35^. This would then prompt consideration of whether or not they would start this medication for this patient, with an associated reassessment of risks vs benefits^35^.

In looking at types of medications, those used to treat symptoms (e.g., benzodiazepines, paracetamol, tramadol) are more often perceived to be inappropriate by clinicians^4^, as well as comparatively easier to deprescribe, when compared to preventative medications^37^. The process of deprescribing is generally initiated to reduce harm in view of side effects, to reduce the pill burden, and to remove medications of minimal or unrealised benefit^22^. There are a number of perceived benefits to deprescribing by clinicians, which include improved patient adherence to therapeutic regimes, cost-effectiveness of treatment plans, and a reduction in health risks such as potential side effects and medication interactions^23^.

## Barriers to deprescribing

There are many perceived barriers to deprescribing, including multiple healthcare providers in both inpatient and outpatient settings, organisational and hierarchical influences, resource limitations, contrasting care expectations, and differing life priorities of patients^33^.

One broad domain that is commonly reported is a lack of knowledge and understanding of the deprescribing process. A deficiency in education and subsequent knowledge of clinicians in deprescribing is commonly reported as a barrier to the process^19,22,23,38–40^. This is compounded by a lack of guidelines targeted towards deprescribing with minimal available evidence to guide practice^24,33,34,36,39^. The difficulty in developing guidelines is likely due, in part, to the fact that polypharmacy can appropriate and beneficial for one patient; however can carry risks of significant harm for another^1,2^. Guidelines are often targeted towards management of a single disease, which can lead to increased polypharmacy^4,22,36–38^. There is also often ambiguity around who is responsible for deprescribing^23^, with clinicians reluctant to undertake this process and shoulder the responsibility of the perceived risks such as worsening symptoms, disease recurrence, drug withdrawal effects, adverse outcomes, shortening life, criticism from both patients and colleagues, as well as damaging relationships and creating inter-disciplinary conflict^20–25,34,41–43^. There is also a distinct concern around the medico-legal implications of the potential risks of deprescribing^33,36,41^.

A second domain in considering barriers to deprescribing is related to health-system factors. The main reported barrier in this domain is the lack of time available for both engaging the patient in the deprescription process and subsequent follow-up^18–20,22–24,33,34,36,38,39,41,43–45^. This process is also not financially viable for many clinicians^39^, particularly in aged care facilities^33^. Another barrier is the absence of a centralised database for patient’s health and medical information^18,39^, in addition to no standardised medicine reconciliation programme^18^. There is also a diffusion of responsibility between doctors^46^, with hospitals typically treating acute issues and generally not viewing deprescribing as their responsibility^46^. This leads to a disruption in continuity of care with multiple clinicians caring for a single patient^18,22,23,33,36,41,42,45^. This lack of continuity^41,43^, along with an increased difficulty of and breakdown of communication between clinicians^20,22–24,33,34,36–39,43–46^, can lead to difficulties in having a single responsible clinician acting as an overarching coordinator of medication management^24,34,36^. This role is typically filled by the general practitioner (GP)^38^, though there can be other practitioners depending upon the patient scenario, for example, nephrologists for patients with kidney failure or kidney transplants. Clinicians often report reluctance to stop medications prescribed by other health practitioners^19,22–25^, especially when the initial indication or planned course of treatment is unclear^20,23,34^. There is also a concern noted by general pracitioners that deprescribed medications can be re-prescribed by specialist physicians, or whilst patients are in hospital^43^.

The last domain involves patient-related factors. There is sometimes resistance from patients to deprescribing, which can be due to a multitude of reasons^21,23,24,39^. Patients, their families, and doctors, have all reported that deprescribing can be seen as an ‘abandonment of care’^4,19,28,37^. It can also be difficult to initiate conversations, especially those related to life expectancy and shifting focus of care from preventative to palliative^37,43^, when for the most part, patients are implicitly satisfied with their current levels of polypharmacy and believe their medications are necessary^21,24,26–32,37,41^. This is in alignment with reports from doctors that patients can have an expectation of prescriptions for medications during consultations^4^. Other common beliefs can be that deprescribing may lead to worsening medical conditions, disease recurrence, adverse consequences, and missing out on future benefits^20,24,25,28,33,47^. Some doctors report that patients may be resistant to deprescribing due to drug dependence, a lack of understanding of the effects of ageing and drug safety, and conflicting messages from physicians^42^. Patients, in turn, report being scared of changing medications, scepticism of the relationship between adverse events and drug therapies, and wanting to know alternatives^28,42^. It can also be difficult to ascertain the degree of adverse effects of polypharmacy experienced by patients as these symptoms are often attributed to ‘older age’^37^. Previous negative experiences with deprescribing are also a significant barrier to engaging with the process again^28,45^. Low levels of health literacy^27,33,45^ and difficulties in communication, compound these above issues^22,41^, and there is a noted lack of educational patient resources tailored to deprescribing^45^. Lastly, a perceived lack of patient motivation in deprescribing^24,33^, combined with doctors not being aware of patient preferences^37^, can ultimately contribute to the option of deprescribing not being explored. (Table 1)

**Table 1:** Key Messages.

Key messages
• Inappropriate polypharmacy is a significant public health challenge.
• Deprescribing is a safe and effective method of managing this issue.
• Deprescribing is a complex and nuanced process in a heterogenous group of patients.
• There are significant barriers such as time, education, and fear of adverse effects.
• To approach deprescribing successfully, there is a need for a collaborative multidisciplinary approach with open and effective communication.

Deprescribing is also perceived to have tangible risks for the patient, versus the intangible benefit of addressing polypharmacy risks. This can then skew the hypothetical risk-benefit ratio for both clinicians and patients^34^. The potential intangible benefit of addressing polypharmacy is also linked to another challenge of deprescribing, which is that patients don’t often present with a recognisable clinical syndrome of polypharmacy, as it is often attributed to unrelated geriatric syndromes, or simply ‘older age’ or chronic ill health^34,37^. This all contributes to a time-consuming process of trying to comprehend unmeasurable “harms vs benefits” in a diverse and complex group of patients with many unknowns^34^, and with both clinical and pharmacological uncertainties^36^.

## Facilitators and enablers of deprescribing

There are a number of factors that can help and enable the deprescribing process, including triggers, opportunities, facilitating influences, and strategies.

‘Triggers’ can be thought of as events that can instigate consideration of deprescribing, and can include side effects or adverse effects linked to medications, evidence of cognitive impairment, diagnosis of a life-limiting disease, functional dependency, and wishes of the patient or their family^19,28,34,45^. Other factors such as number of medications, age of the patient, number of comorbidities, and budgetary constraints can also be considered triggers^19,32,36,41^.

There are a number of recognised opportunities to prompt a routine medication review and potential deprescribing, including the transition of care between clinicians, admissions to nursing homes, upon regular intervals i.e., 6 or 12 monthly, and patient factors^25^. These patient factors include patient’s asking about their medications, belief that they are no longer required or causing side effects, ability to manage their own health, and wanting to reduce the number of medications that they are taking^4,20,28,30,39^.

The use of regular strategies can also help facilitate the deprescribing process, such as using a gradual approach to changing medication regimes, consider deprescribing during hospital admissions if feasible, involve specialists to help mitigate uncertainty, patient education and involvement in choosing whether to deprescribe and selecting ‘easier’ medications to deprescribe (i.e., statins or complementary medications)^34,43,46^. This last strategy is, however recognised as not addressing some high-risk medications such as anti-thrombotic, opioid, or psychotropic drugs^34^. Other facilitating influences that have been described include education of patients and family around what deprescribing is, the risks of inappropriate polypharmacy, and what alternative non-pharmacological strategies are available^18,22,27,32,34,36,41,42,45^. This is a key area for future focus as health literacy amongst patients has a positive correlation with willingness to deprescribe medications^27^. Another key area is the education and training of clinicians in deprescribing^24^. Senior doctors with more experience are generally more comfortable with deprescribing^38^, and many doctors cite training and education as key enabling factors^18,33,39,40,42,43^. The production of evidence and development of evidence-based guidelines has also been noted as a main enabling factor^22,23,34,38–41,43^.

The use of a multidisciplinary team approach to deprescribing, especially with the involvement of pharmacists in medication reviews, is a key strategy for success^18,20,22,23,34,36,41,45^. This implicitly involves improving the lines of communication between the multiple clinicians providing care for each patient^20,22,36,39–41,43–46^. Building strong relationships between clinicians and patients, with continuity of care, helps to facilitate this process^18,29,39,42,43,45^.

Finally, one of the key facilitating influences described on successfully deprescribing is having adequate time allocated for the entire medication review process, including deprescribing and opportunity for follow-up as required^20,23,27,38,39^. Whilst requiring a commitment of time, resources, and financial support^23,39,40,43^ there is likelihood of this being substantially offset if not justified by decreased overall health service utilisation.

## Future directions in polypharmacy and deprescribing

Education, of both clinicians and patients, is widely recognised as a key enabling factor in addressing polypharmacy by deprescribing^4,18,22,23,34,39,45^. This involves reframing risk perceptions and highlighting the fact that polypharmacy has a risk just like any medical condition, and this risk can increase with increasing age^34^. This may involve a change in mindset for both clinicians and patients^22^.

For clinicians, increasing the awareness of students and earlier career clinicians to the risks of polypharmacy and the benefits of deprescribing is essential^24^, as is improving curricular education during training^40^. Continuing professional development^33^ in addition to interprofessional education and collaboration in postgraduate years is also vital^18,20,36,38^, such as regular multidisciplinary and case-based discussions^36^. This can take the form of workshops targeted towards how to effectively deprescribe^24^ in addition to reflective practice. Raising awareness of cognitive biases present in clinicians that prevent deprescribing has also been identified as an important area^34^. The increase in knowledge and awareness of polypharmacy can also lead to improved recognition of adverse drug events, which can trigger medication reviews and addressing high-risk medications^33^.

With respect to patient education, it is important to ensure the provision of appropriate medication counselling that involves providing information such as indications, side effects, precautions, drug-drug interactions, as well as deprescribing options^18,22,28^. It is also vital to provide clear information on the risk:benefit ratio of medication for patients^37^, as well as how this ratio may change with time^34,42^. This may require a restructuring of appointments to allow for discussion around concerns with medications and involvement in decision-making processes^27^. Provision of educational material in a variety of tools such as online, printed, and interactive forms can also be beneficial^18^. Education is important as improving health literacy is associated with willingness to deprescribe^27^, as is establishing patients’ trust in prescribing and medications^32^.

Maintaining both continuity of care^18,29^ and improving the lines of communication of both provider-provider and provider-patient is fundamental to addressing inappropriate polypharmacy^18,20,23,34,36,39,43,44,46^. This also assists in building strong relationships with patients, especially those experiencing complex multimorbidities and polypharmacy, and helps facilitate shared-decision-making^26,34,37,43,46^, and the subsequent ability to successfully deprescribe^28,29,42,45^.

Identifying those at risk of adverse effects of polypharmacy is essential^29^, which requires appropriate time to be allocated for comprehensive, routine, and frequent health assessments, which include medication reviews to screen for inappropriate polypharmacy^22,24,33,34,38^. It is useful to ask about the subjective burden of medications^28^ as well as use objective measures, such as number of medications^36^, to help facilitate these reviews. Subsequent discussion around risk-benefit profiles of medications then needs to have a person-centered focus^43^. Having these regular and early discussions around medication management that is guided by evidence^42^, with documentation of the patient’s preferences and ‘goals of care’, can improve decision-making and person-centred care throughout the lifespan^21,37,43^. Discussing the beliefs and attitudes of patients is important to ascertain any barriers towards deprescribing, as is having proactive conversations around the modification of targets for chronic disease management as well as reducing medication burden^37^.

A multidisciplinary and collaboration approach with effective communication is essential to successful deprescribing^20,23,36,39,40,44–46^. This would include ease of access to patient records, ideally electronic records, and comprehensive discharge summaries with appropriate information about the prescribing and deprescribing of medications^39–41,43–45^. Integrated care teams for each patient with the involvement of pharmacists and appropriate specialists are essential^18,20,22,23,36^. Seeking support for deprescribing from specialists can assist GPs and primary care providers in decision-making processes^38^. Pharmacists can perform key roles such as medication reconciliation, consultations with patients to identify adverse drug events, compliance issue identification, adherence counselling, and provide recommendations as to appropriateness of medications and suggestions of deprescribing when applicable^20,22,34,41,45^. Other vital roles of pharmacists include prescription screening, provision of information, assessment of medication adherence, and medication counselling^22,41^.

Improving access to user-friendly and easily accessible deprescribing tools such as aids or algorithms can assist in deprescribing^20,22,24,34,36,41^, in addition to having evidence-based process guidelines^22,38,39,41,43^. The availability of better evidence to support when deprescribing is safe and effective can assist in both decision-making processes^43^ and the development of standard protocols for medication management^33,34,43^. Structured dialogue to help facilitate communication about the risks and benefits of deprescribing has also been identified as a useful tool^28,43^. When deprescribing, implementing gradual withdrawal of individual medications^34,43^, allowing for restarting of medications if condition or symptoms return, and associated ongoing monitoring with good communication is essential^43^. It is also important to be able to provide clear instructions for patients on how to cease or reduce their medication dosages^28^. However, it is also important to recognise that due to the heterogeneity of patients, it is unlikely that a singular comprehensive management of polypharmacy guideline could be designed^37^. Individualisation is a central and practical tenet.

Moving forward in addressing polypharmacy, it is important to recognise that the majority of patients are willing to reduce the number of medications that they take^26–32^, however, patients can vary in the extent to which they are involved in the decision-making process^30^. Therefore, opportunities for medication reviews and subsequent deprescribing, when appropriate, should always be taken^30^.

Recognised limitations of this review include the inherent bias associated with narrative reviews with the selection of literature. This bias has been mitigated but not removed through the semi-systematic approach outlined in our PRISMA flowchart (Supplementary Fig. 1). It is also noted that polypharmacy has variable definitions, with the one used in this review being one of the most commonly accepted, i.e., concurrent use of ≥5 medications being used. The inclusion of supplements, over-the-counter medications, and traditional and complementary medicines in the count for polypharmacy also increases the difficulty in finding the true values of polypharmacy in a population. Deprescribing outcomes are also heterogenous in nature throughout the literature, with individual interpretations of what is a ‘successful’ outcome might be defined as either qualitatively or quantitatively (e.g., drug cessation, dose reduction, reduction in adverse events).

In conclusion, polypharmacy in older patients is substantially prevalent and deprescribing involves nuanced decision-making in a complex and heterogenous group of patients^34^. There are many barriers to deprescribing, ranging from lack of time and confidence in implementing the process, to fear of the unknown. It is essential to maintain good communication between healthcare providers as well as with patients^39^. Strong relationships built on trust and transparency are vital in maintaining person-centred care and reducing the risks of polypharmacy in addition to addressing it^39^.

### Reporting summary

Further information on research design is available in the Nature Research Reporting Summary linked to this article.

### Supplementary information

Supplementary

Reporting Summary
