# Supplementary material for: Attitudes and barriers towards deprescribing in older patients experiencing polypharmacy: a narrative review
Source: NPJ Aging. 2024 Jan 23;10(1):6. doi: 10.1038/s41514-023-00132-2 (PMC10806180; doi:10.1038/s41514-023-00132-2)
Supplement: Supplementary file 1 — Supplementary [file 41514_2023_132_MOESM1_ESM.docx]

**Supplementary Methods**

Search Strategies Utilised

MEDLINE (Ovid)

| 1 | Deprescriptions/ |
| --- | --- |
| 2 | Drug tapering/ |
| 3 | Polypharmacy/ |
| 4 | Inappropriate prescribing/ |
| 5 | Potentially inappropriate medication list/ |
| 6 | Medical overuse/ |
| 7 | Overtreatment/ |
| 8 | Aged/ |
| 9 | Aged, 80 and over/ |
| 10 | Frail elderly/ |
| 11 | Geriatrics/ |
| 12 | Geroscience/ |
| 13 | 1 OR 2 |
| 14 | 3 OR 4 OR 5 OR 6 OR 7 |
| 15 | 8 OR 9 OR 10 OR 11 OR 12 |
| 16 | 13 AND 14 AND 15  English language  Humans  2012-2022 |

CINAHL (EBSCO)

| 1 | Deprescribing |
| --- | --- |
| 2 | Drug tapering |
| 3 | Polypharmacy |
| 4 | Inappropriate prescribing |
| 5 | Aged |
| 6 | Aged, 80 and over |
| 7 | Frail elderly |
| 8 | Gerontologic care |
| 9 | 1 OR 2 |
| 10 | 3 OR 4 |
| 11 | 5 OR 6 OR 7 OR 8 |
| 9 | 9 AND 10 AND 11  English language  Humans  2012-2022 |

EMCARE (OVID)

| 1 | Deprescription/ |
| --- | --- |
| 2 | Drug dose reduction/ |
| 3 | Polypharmacy/ |
| 4 | Inappropriate prescribing/ |
| 5 | Potentially inappropriate medication/ |
| 6 | Aged/ |
| 7 | Very elderly/ |
| 8 | Frail elderly/ |
| 9 | Geriatrics/ |
| 10 | Gerontology/ |
| 11 | Geriatric care/ |
| 12 | 1 OR 2 |
| 13 | 3 OR 4 OR 5 |
| 14 | 6 OR 7 OR 8 OR 9 OR 10 OR 11 |
| 15 | 12 AND 13 AND 14  English language  Humans  2012-2022 |

SCOPUS

| 1 | Deprescrib* OR deprescrip* OR “de-prescription” OR “deprescriptions” |
| --- | --- |
| 2 | “drug tapering” OR “dose reduction” OR “dose reductions” OR “dose tapering” OR “dose taperings” OR “drug tapering” OR “reduction, dose” OR “reductions, dose” OR “tapering, dose” OR “tapering, drug” OR “taperings, dose” OR “dose reduction” OR “dose tapering” OR “drug weaning” OR “drug dose reduction” OR “dosage decrease” OR “dosage reduction” OR “dose decrease” OR “dose reduction” OR “drug tapering” OR “reduction of drug dosage” OR “reduction of drug dose” OR “reduction, drug dose” |
| 3 | Polypharmacy OR polymedication OR “multi-drug therapy” OR “multidrug therapy” OR “multiple drug therap*” OR “multiple drug treatment” OR “multiple pharmacotherapy” OR “pharmacotherapy, multiple” OR “pharmacy, poly” OR “poly pharmacy” OR “polypragmasia” OR “polypragmasy” |
| 4 | “medical overuse” OR “health care, unnecessary” OR “health services overuse” OR “health services overuses” OR “health services overutilization” OR “medical care, unwanted” OR “medical overuse” OR “medical overuses” OR “medical preference misdiagnoses” OR “medical preference misdiagnosis” OR “misdiagnosis, medical preference” OR “misdiagnosis, preference” OR “overuse, health services” OR “overuse, medical” OR “overuses, health services” OR “overuses, medical” OR “overutilization, health services” OR “overutilization of health services” OR “preference misdiagnoses” OR “preference misdiagnosis” OR “unnecessary health care” OR “unwanted medical care” |
| 5 | “over prescrib*” OR “overtreatment” OR “over treatment” OR “over-treatment” OR “over-treatments” OR “overtreatment” OR “overtreatments” |
| 6 | “potentially inappropriate medication*” OR “medication appropriateness index” OR “potentially inappropriate medication list” OR “potentially inappropriate prescrib*” OR “potentially inappropriate prescrip*” OR “inappropriate prescrib*” OR “inappropriate prescrip*” OR “prescribing, inappropriate” OR “prescribing, over” OR “prescribings, inappropriate” OR “prescribings, over” OR “prescription, inappropriate” OR “prescriptions, inappropriate” OR “potentially inappropriate medication list” OR “appropriateness index, medication” OR “appropriateness indices, medication” OR “beers criteria” OR “beers criteria, de” OR “criteria, stopp start” OR “criterias, stopp start” OR “inappropriate medication, potentially” OR “inappropriate medications, potentially” OR “index, medication appropriateness” OR “indices, medication appropriateness” OR “medication appropriateness index” OR “medication appropriateness indices” OR “medication, potentially inappropriate” OR “medications, potentially inappropriate” OR “pim list” OR “pim lists” OR “stopp” OR “stopp (screening tool of older person's potentially inappropriate prescriptions) ” OR “screening tool of older person's potentially inappropriate prescriptions” OR “de beers criteria” |
| 7 | Aged OR elderly OR “aged, 80 and over” OR “oldest old” OR “Elderly, 80 and over” OR “Over 80” OR “Aged patient” OR “Aged people” OR “Aged person” OR “Aged subject” OR “Elderly patient” OR “Elderly people” OR “Elderly person” OR “Elderly subject” OR “Senior citizen” OR “Senium” OR “very elderly” OR “aged, 80 and over” OR centenarian OR centenarians OR nonagenarian OR nonagenarians OR octogenarian OR octogenarians OR “very old” |
| 8 | “frail elderly” OR “adult, frail older” OR “adults, frail older” OR “elder, frail” OR “elderly, frail” OR “elderly, functionally-impaired” OR “elders, frail” OR “frail elder” OR “frail elderly” OR “frail elders” OR “frail older adult” OR “frail older adults” OR “functionally impaired elderly” OR “functionally-impaired elderly” OR “older adult, frail” OR “older adults, frail” OR “Frail older adult” OR “Frail older adults” |
| 9 | “geriatrics” OR “gerontology” OR “geroscience” OR “old persons” OR “older persons” OR “80 and over, aged” OR “80 and over” OR “Aged over 80” OR “gerontologic care” OR “aged care” OR “geriatric care” OR “gerontological care” OR “geriatric aspects” OR “geriatric medicine” OR “geriatric practice” OR geriatry OR sociogeriatrics OR “geriatric care” OR “gerontologic care” |
| 10 | 1 OR 2 |
| 11 | 3 OR 4 OR 5 OR 6 |
| 12 | 7 OR 8 OR 9 |
| 13 | 10 AND 11 AND 12  English language  Human  2012-2022 |

**Supplementary Figure 1. Identification of literature**

**Identification of studies via databases and registers**

Records removed *before screening*:

Duplicate records removed (n = 389)

Records identified from*:

CINAHL (n = 58)

EMCARE (n = 111)

MEDLINE (n = 279)

SCOPUS (n = 1006)

**Identification**

Records screened

(n = 1065)

Records excluded**

(n = 980)

Reports sought for retrieval

(n = 85)

Reports not retrieved

(n = 4)

**Screening**

Reports assessed for eligibility

(n = 81)

Reports excluded:

Reason 1 (n = 19)

Reason 2 (n = 11)

Reason 3 (n = 2)

Reason 4 (n = 16)

Reason 5 (n = 2)

Studies included in review

(n = 31)

Reports of included studies

(n = 31)

**Included**

Reason 1 – participants taking <5 medications

Reason 2 – participants aged <65 years

Reason 3 – study refers to specific comorbidities

Reason 4 – study does not discuss barriers to deprescribing

Reason 5 – incorrect study format (see inclusion/exclusion criteria)
